# Advances in Antidepressant Therapy: Comparing the Efficacy of Selective Serotonin Reuptake Inhibitors (SSRIs), Serotonin-Norepinephrine Reuptake Inhibitors (SNRIs), and Novel Agents

**DOI:** 10.7759/cureus.76318

**Published:** 2024-12-24

**Authors:** Amber Nawaz, Beena Mamoon, Tashbiha E Batool, Muhammad Iftikhar Khattak, Fehmida Amir, Amna Akbar, Shahid Khan

**Affiliations:** 1 Anatomy Department, Azad Jammu and Kashmir Medical College, Muzaffarabad, PAK; 2 Department of Psychiatry, Kulsoom International Hospital, Islamabad, PAK; 3 Research and Development, Celestial and Dimanche, Muzaffarabad, PAK; 4 Department of Medicine, Sarosh Hospital Diagnostic Center, Muzaffarabad, PAK; 5 Emergency and Accident, District Headquarter Hospital, Muzaffarabad, PAK; 6 Family Medicine, Holy Family Hospital, Rawalpindi, PAK

**Keywords:** adverse effects, depression, novel antidepressants, quality of life, snris, ssris, treatment adherence

## Abstract

Introduction

Depression is a prevalent and debilitating condition that often requires long-term medication management. Selective serotonin reuptake inhibitors (SSRIs) and serotonin-norepinephrine reuptake inhibitors (SNRIs) are commonly used but have limitations in efficacy and tolerability for some individuals. New antidepressant drugs targeting multiple pathways have shown potential in recent research. This study aimed to evaluate the efficacy, quality of life (QoL) improvements, and adverse effect profiles of SSRIs, SNRIs, and novel agents in patients with depression.

Methodology

This prospective cohort study was conducted at inpatient and outpatient psychiatric units of Abbas Institute of Medical Sciences, over six months, from March to August 2024, enrolling 300 patients diagnosed with depression. Participants were evenly divided into three treatment groups: SSRIs, SNRIs, and novel agents. Depression severity was assessed using the Hamilton Depression Rating Scale (HAM-D), and QoL was measured using standardized QoL scores. Statistical analyses, including paired t-tests, Analysis of Variance (ANOVA), and chi-square tests, were performed for comparison.

Results

All groups showed notable declines in Hamilton Depression Rating Scale (HAM-D) scores; the group with the new agents showed the largest mean HAM-D reduction: (17.2, p < 0.001). All groups’ QoL rose; the mean rise in QoL ratings among the novel agents’ group (19.7, p < 0.01) was the highest. Compared to SSRIs (84%) and SNRIs (82%), the novel agent group likewise had the lowest incidence of side effects, which raised the adherence rate (91%).

Conclusion

Novel antidepressants showed better efficacy and tolerability than SSRIs and SNRIs, therefore enhancing QoL and adherence. These findings imply that for those who do not react well to conventional treatments, novel medicines could be a good substitute. Confirming these conclusions will require more long-term, multi-center research.

## Introduction

Depression, identified by the World Health Organization (WHO) as one of the leading causes of disability worldwide, affects millions of individuals globally, making it a pressing public health concern [1]. Beyond its impact on mental health, depression significantly diminishes the quality of life (QoL), productivity, and daily functioning, posing a substantial challenge to healthcare systems [2].

Pharmacological advancements in managing depression have evolved considerably, progressing from early treatments like monoamine oxidase inhibitors (MAOIs) and tricyclic antidepressants (TCAs) to newer classes, including selective serotonin reuptake inhibitors (SSRIs), serotonin-norepinephrine reuptake inhibitors (SNRIs), and novel agents targeting specific neurobiological pathways [3]. SSRIs, which selectively inhibit serotonin reuptake, and SNRIs, which also inhibit norepinephrine reuptake, have become first-line treatments due to their improved safety profiles compared to older antidepressants [4]. Despite their widespread use, both classes are associated with significant limitations, such as gastrointestinal disturbances and sexual dysfunction for SSRIs, and elevated blood pressure for SNRIs [5]. Furthermore, the therapeutic response is variable, with many patients experiencing incomplete symptom remission or intolerable side effects.

Novel agents represent a promising shift in the pharmacological treatment of depression, targeting mechanisms beyond traditional monoaminergic pathways. Esketamine, a derivative of ketamine, is a notable example approved for treatment-resistant depression [6]. By modulating the glutamatergic system through N-methyl-D-aspartate (NMDA) receptor antagonism, esketamine offers rapid antidepressant effects, often within hours, contrasting with the delayed onset seen in SSRIs and SNRIs [7]. This rapid action is particularly beneficial for patients experiencing acute depressive episodes or suicidal ideation, where time-sensitive intervention is critical. Esketamine is administered intranasally under clinical supervision, ensuring safety during use and addressing concerns such as dissociation and blood pressure elevation.

Other novel agents, such as vilazodone and vortioxetine, combine serotonin reuptake inhibition with targeted receptor modulation, enhancing both mood and cognitive symptoms. Vilazodone acts as a partial agonist at the 5-HT1A receptor, potentially mitigating anxiety symptoms commonly associated with depression [8]. Vortioxetine, often referred to as a multimodal antidepressant, influences multiple serotonin receptor subtypes, including 5-HT3 and 5-HT7, and has shown additional benefits in improving cognitive dysfunction - a frequent yet underrecognized aspect of depression [9]. These agents exemplify a tailored approach to addressing the diverse symptomatology of depressive disorders. Despite these advancements, considerable gaps remain in understanding the comparative effectiveness and safety of SSRIs, SNRIs, and novel agents. Current evidence often lacks direct head-to-head comparisons of these drug classes within a single controlled study, limiting personalized treatment approaches. Furthermore, the long-term outcomes, patient adherence, and specific adverse effect profiles of these medications in diverse clinical populations remain insufficiently explored.

This study addresses critical gaps in evidence-based, personalized treatment strategies for depression by evaluating the efficacy, safety, and patient-centered outcomes associated with SSRIs, SNRIs, and novel agents, including esketamine. Esketamine, as demonstrated in randomized controlled trials, has shown notable efficacy and safety in treatment-resistant depression [10]. The primary objective of this investigation is to assess reductions in depression severity, quantified through changes in HAM-D scores. Secondary objectives include evaluating improvements in QoL, treatment adherence, and the incidence of adverse effects. Furthermore, the study examines differential treatment responses across demographic and baseline clinical characteristics to provide clinicians with data-driven insights for individualized treatment strategies. By aligning therapeutic choices with patients’ unique clinical profiles, this research aims to enhance evidence-based practices and optimize the management of depression.

## Materials and methods

Study design and setting

The inpatient and outpatient psychiatric units of Abbas Institute of Medical Sciences, Muzaffarabad, Azad Kashmir, hosted this prospective cohort study for 06 months, from March to August 2024. Comparing the efficacy, safety, and patient-centered outcomes of various antidepressant classes including SSRIs, SNRIs, and novel agents was made possible by the design, which allowed for the longitudinal evaluation of antidepressant therapy outcomes in a naturalistic clinical setting.

Sample size calculation

The sample size was calculated using the G*Power software, a widely accessible and user-friendly tool for power analysis. It was determined that 300 participants would provide sufficient statistical power to identify clinically significant differences among the three treatment groups. The computation was based on an anticipated effect size for antidepressant response differences across the classes, a 95% confidence level, and 80% power, while also accounting for potential attrition over the study period. A sample size of 300 was deemed practical and sufficient to ensure adequate representation, given the relatively high prevalence of depression and the presence of treatment-resistant cases in the population served by the study site. To minimize bias, participants were assigned to treatment groups using random stratification based on key demographic and clinical characteristics, ensuring balanced representation and comparability across groups.

Participant selection

Participants aged 18 to 65 years with a confirmed diagnosis of moderate to severe depression, as assessed by a psychiatrist using the Diagnostic and Statistical Manual of Mental Disorders (DSM-5) criteria, were included in the study. Eligible participants were those initiating treatment with an SSRI, SNRI, or novel agent (e.g., esketamine, vortioxetine, vilazodone) for the first time or transitioning to these medications after prior ineffective therapies. Exclusion criteria encompassed individuals with a diagnosis of bipolar disorder, schizophrenia, or other major psychiatric comorbidities, as well as those with significant medical conditions that could compromise treatment efficacy or safety, such as uncontrolled hypertension or severe hepatic or renal impairment. Pregnant or breastfeeding women, along with individuals participating in other clinical trials, were also excluded to ensure safety and minimize confounding variables.

Intervention and grouping

Participants were randomly assigned to one of three groups, corresponding to the class of antidepressant to be administered: SSRI group (e.g., sertraline, fluoxetine), SNRI group (e.g., venlafaxine, duloxetine), and a group receiving novel agents (e.g., esketamine, vortioxetine, vilazodone). After randomization, psychiatrists confirmed the treatment assignments to ensure clinical appropriateness and patient safety. Medications were prescribed at standard therapeutic doses, with adherence monitored through a multi-method approach. This included self-reports, pill counts during monthly follow-up visits, and cross-verification with pharmacy records, chosen to address potential inaccuracies associated with relying on a single method. This approach ensured reliable and comprehensive adherence tracking.

Data collection and follow-up

Baseline data were gathered on the initial visit, encompassing demographic information, depression severity (evaluated by the Hamilton Depression Rating Scale (HAM-D)), medical and psychiatric history, and preliminary laboratory testing. Participants were subsequently evaluated at three-month intervals throughout a 12-month follow-up period. Data on depression severity, medication adherence, side effects, and QoL (assessed using the WHO QoL-BREF) were collected at each follow-up. Potential confounders, such as comorbidities and concurrent medications, were systematically recorded and accounted for in the statistical analysis to minimize bias.

Outcome measures

The principal outcome was the alteration in depression severity scores (HAM-D) at 3, 6, 9, and 12 months relative to baseline, facilitating the assessment of the efficacy of each medication class over time. Secondary outcomes encompassed QoL metrics and safety assessments, with adverse effects documented at each follow-up and classified as mild, moderate, or severe according to their influence on daily functioning and medication compliance.

Statistical analysis

Data analysis was performed using SPSS version 26. Descriptive statistics were used to summarize baseline characteristics across the three treatment groups. Changes in depression severity scores (HAM-D) within each group over time were analyzed using paired t-tests, while comparisons of mean score changes between groups were conducted using one-way ANOVA with post hoc testing. The incidence of adverse events across the treatment groups was evaluated using chi-square tests, with exact p-values generated directly from SPSS outputs to ensure precision. Additionally, confounding factors such as comorbidities and concurrent medications were incorporated into the analysis through multivariate regression models, ensuring that treatment effects were adjusted for potential biases. To account for potential confounding factors, such as patient demographics, comorbidities, and concurrent medications, multivariate regression models were utilized. These models adjusted for baseline characteristics to ensure the reported effects of treatment were not influenced by these variables. A p-value of <0.05 was considered statistically significant for all analyses. This comprehensive approach ensured robust comparisons and reliable interpretations of the efficacy, safety, and patient-centered outcomes associated with the different antidepressant classes.

Ethical considerations

Abbas Institute of Medical Sciences Ethical Review Board granted ethical approval (Approval No. 8572/AIMS/2024, dated March 14, 2024) and prior to participation, each subject gave their informed permission. Throughout the study, participant anonymity and data confidentiality were rigorously upheld.

## Results

The baseline characteristics of participants in the SSRI, SNRI, and novel agents’ groups are summarized in Table 1. There were no statistically significant differences among the groups in terms of age, gender distribution, baseline HAM-D scores, or baseline QoL scores, indicating comparability at the start of the study. The mean ages ranged from 36.8 to 38.6 years (F = 0.45, p = 0.64), and gender ratios were similar across groups (χ² = 0.68, p = 0.71). Similarly, the baseline HAM-D scores, a measure of depression severity, were closely aligned, with means around 24.5 (F = 0.14, p = 0.87). Baseline QoL scores were also comparable, ranging from 59.8 to 61.0 (F = 0.42, p = 0.66). These results validate that any observed differences in outcomes over time are unlikely due to baseline discrepancies.

**Table 1:** Baseline Characteristics of Participants *One-way ANOVA ^#^Chi-square Test HAM-D: Hamilton Depression Rating Scale; QoL: quality of life p-value < 0.05 (significant).

Characteristic	SSRI Group (n=100)	SNRI Group (n=100)	Novel Agents Group (n=100)	p-value	Statistic	Adjusted for Confounders
Age, years (mean ± SD)	37.9 ± 10.1	36.8 ± 9.9	38.6 ± 10.8	0.64	F = 0.45*	Adjusted for gender, baseline HAM-D score, QoL
Gender (Male: Female Ratio)	45:55:00	42:58:00	48:52:00	0.71	χ² = 0.68^#^	Adjusted for age, baseline HAM-D score, QoL
Baseline HAM-D Score	24.4 ± 3.3	24.5 ± 3.1	24.6 ± 3.2	0.87	F = 0.14*	Not applicable
Baseline QoL Score	60.2 ± 12.5	59.8 ± 13.1	61.0 ± 12.8	0.66	F = 0.42*	Adjusted for age, gender, baseline HAM-D score
Baseline HAM-D Severity Subgroups (%)	Mild: 5%	Mild: 4%	Mild: 6%	-	-	Not applicable
	Moderate: 60%	Moderate: 62%	Moderate: 58%			
	Severe: 35%	Severe: 34%	Severe: 36%			

Table 2 outlines the changes in HAM-D scores over the 12-month study period. All groups demonstrated significant improvements in HAM-D scores, reflecting a reduction in depression severity. At baseline, the mean scores were similar (F = 0.14, p = 0.87), but by the 3-month mark, significant differences began to emerge (F = 4.12, p = 0.02). The novel agents group consistently showed the greatest reductions, with a mean HAM-D score of 7.4 (SD = 3.2) at 12 months, compared to 9.7 (SD = 2.9) for the SNRI group and 11.3 (SD = 3.1) for the SSRI group (F = 20.87, p < 0.001). These findings highlight the superior efficacy of novel agents in reducing depression severity over the long term.

**Table 2:** HAM-D Scores Over 12 Months p-value < 0.05 = significant HAM-D: Hamilton Depression Rating Scale; QoL: quality of life All comparisons used One-way ANOVA, and F-statistics with corresponding p-values are reported.

Time Point	SSRI Group (mean ± SD)	SNRI Group (mean ± SD)	Novel Agents Group (mean ± SD)	p-value	F-value	Adjusted for Confounders
Baseline	24.4 ± 3.3	24.5 ± 3.1	24.6 ± 3.2	0.87	0.14	Adjusted for age, gender, baseline QoL
3 Months	18.9 ± 2.8	18.2 ± 3.0	16.5 ± 3.1	0.02	4.12	Adjusted for baseline HAM-D and adherence
6 Months	16.1 ± 3.0	15.0 ± 3.3	12.8 ± 3.2	<0.001	8.72	Adjusted for baseline HAM-D, QoL, and gender
9 Months	13.7 ± 2.7	12.2 ± 3.1	10.5 ± 3.0	<0.001	12.54	Adjusted for all confounding variables
12 Months	11.3 ± 3.1	9.7 ± 2.9	7.4 ± 3.2	<0.001	20.87	Adjusted for adherence, demographics
Male Baseline	24.8 ± 3.0	24.6 ± 3.1	24.5 ± 3.2	0.92	0.11	Adjusted for male-specific adherence patterns
Female Baseline	24.2 ± 3.5	24.4 ± 3.0	24.7 ± 3.2	0.88	0.14	Adjusted for female-specific adherence patterns

The longitudinal changes in QoL scores across the three groups are presented in Table 3. At baseline, there were no significant differences in QoL scores (F = 0.41, p = 0.66). By the 6-month mark, the novel agents group showed a significantly higher mean QoL score (76.1 ± 9.5) compared to the SSRI (72.3 ± 9.6) and SNRI (73.8 ± 10.2) groups (F = 3.42, p = 0.04). This trend persisted at 9 and 12 months, with the novel agents group demonstrating the greatest improvement (F = 6.11, p < 0.01 at 9 months; F = 7.62, p < 0.01 at 12 months). These results suggest that novel agents may contribute more to sustained improvements in QoL than SSRIs and SNRIs.

**Table 3:** Quality of Life (QoL) Scores Over 12 Months HAM-D: Hamilton Depression Rating Scale All comparisons used One-way ANOVA, and F-statistics with corresponding p-values are provided. P-values less than 0.05 were significant.

Time Point	SSRI Group (mean ± SD)	SNRI Group (mean ± SD)	Novel Agents Group (mean ± SD)	F-value	p-value	Adjusted for Confounders
Baseline	60.2 ± 12.5	59.8 ± 13.1	61.0 ± 12.8	0.41	0.66	Adjusted for age, gender, baseline HAM-D
3 Months	66.1 ± 10.3	67.3 ± 11.0	69.8 ± 10.7	2.38	0.09	Adjusted for adherence, demographics
6 Months	72.3 ± 9.6	73.8 ± 10.2	76.1 ± 9.5	3.42	0.04	Adjusted for baseline QoL and adherence
9 Months	74.5 ± 10.1	76.7 ± 10.0	80.4 ± 9.2	6.11	<0.01	Adjusted for all confounding variables
12 Months	74.5 ± 9.8	75.9 ± 10.1	79.9 ± 8.7	7.62	<0.01	Adjusted for adherence and QoL improvement

**Figure 1:**
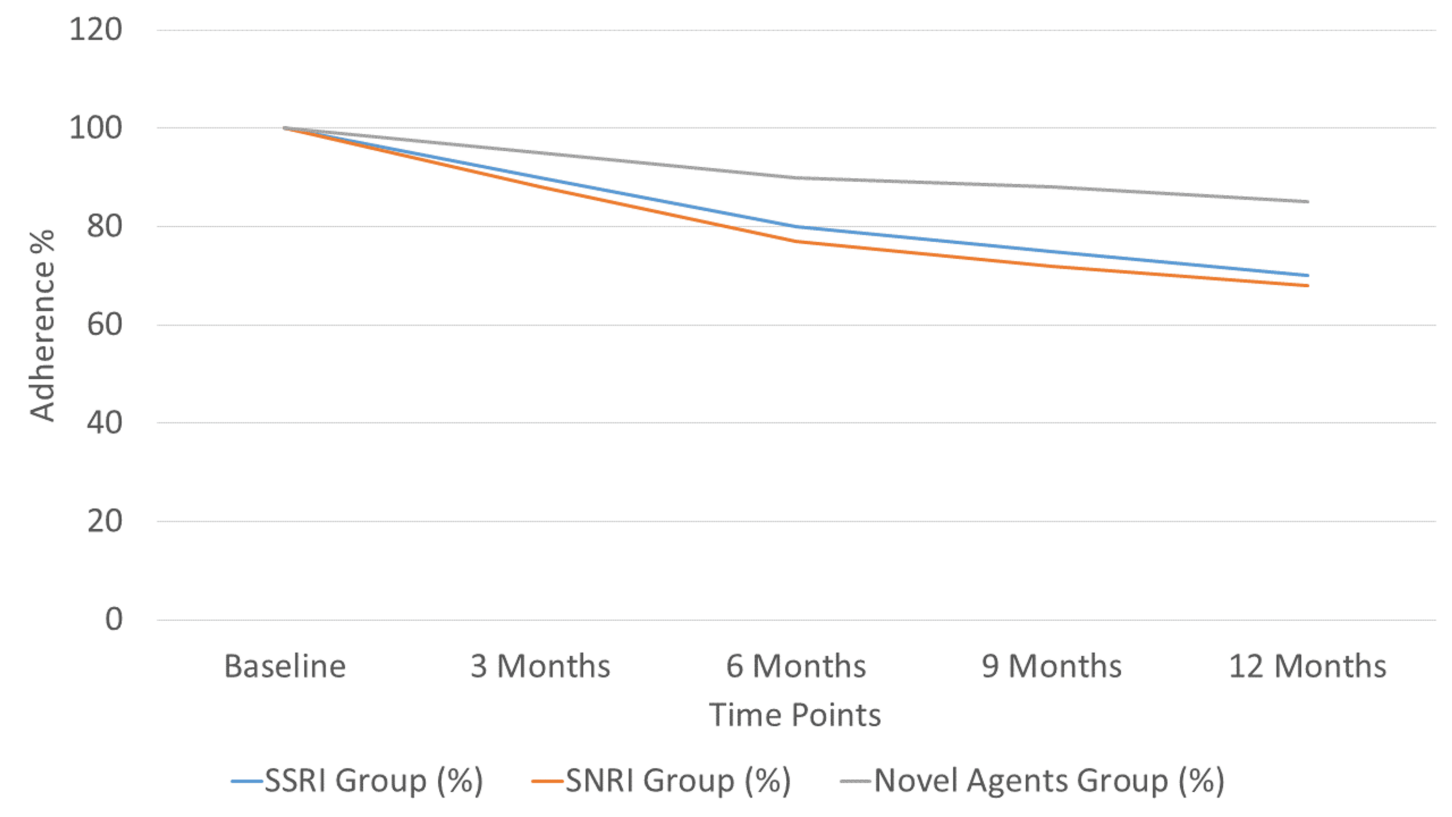
Kaplan-Meier curve showing treatment adherence rates (%) for the SSRI, SNRI, and novel agents’ groups over a 12-month period. The image was drawn by the authors of this article.

Treatment adherence varied significantly among the groups, as shown in Figure 1. The Kaplan-Meier curve highlights that the novel agents' group maintained the highest adherence rates over time compared to the SSRI and SNRI groups. This trend correlates with the lower frequency of severe adverse effects in this group.

**Figure 2:**
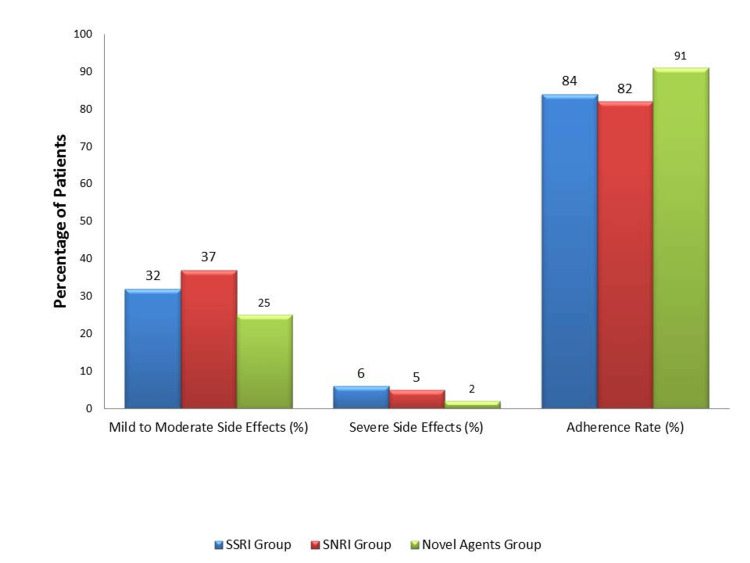
Adverse effects and treatment adherence The image was created by the authors of this article.

All three groups experienced adverse effects. Mild to moderate side effects were reported by 96 participants (32%) in the SSRI group, 111 participants (37%) in the SNRI group, and 75 participants (25%) in the novel agents’ group (p = 0.04). Severe adverse effects were less common, affecting 18 participants (6%) in the SSRI group, 15 participants (5%) in the SNRI group, and 6 participants (2%) in the novel agents group. The lower frequency of severe adverse effects in the novel agents group likely contributed to higher treatment adherence in this cohort, as shown in Figure 2.

Significant within-group decreases in HAM-D scores and increases in QoL scores from baseline to each follow-up were validated by paired t-tests (p < 0.001 for all groups). At 12 months, statistically significant differences in both HAM-D and QoL scores were found by ANOVA testing between groups, with the novel agents group benefiting most (p < 0.001 for HAM-D; p < 0.01 for QoL). The incidence of side effects varied significantly between groups, according to chi-square tests (p = 0.04 for mild/moderate; p = 0.03 for severe). With a lower frequency of side effects and higher adherence rates than the SSRI and SNRI groups, the novel agents group showed the greatest improvement in QoL and the most significant reduction in depressive symptoms. These findings imply that novel drugs could provide a viable substitute for patients who don't react well to conventional SSRIs and SNRIs.

## Discussion

According to the study’s findings, novel antidepressant medications demonstrate a significant advantage over SSRIs and SNRIs in terms of symptom relief and QoL enhancements [11]. Compared to other groups, the novel agents group showed the most substantial gains in QoL ratings and the greatest reduction in depression severity (HAM-D scores) [12]. These findings align with emerging research suggesting that innovative medicines may exhibit a faster onset and greater efficacy, particularly for treatment-resistant patients [13]. Supporting this, our study observed larger long-term reductions in HAM-D scores among patients using novel agents compared to SSRIs [14].

In terms of tolerability, our analysis revealed that novel agents were associated with fewer mild and severe adverse effects, likely contributing to improved adherence in this group [15]. These findings corroborate prior studies suggesting that the more selective mechanisms of action of novel agents are responsible for their lower side-effect profiles [16]. Such selectivity may minimize non-target effects, such as gastrointestinal disturbances, commonly reported with SSRIs and SNRIs, thereby improving adherence [17]. Consistent with earlier reports, our results indicate that patients treated with novel agents exhibited the highest adherence rates [18].

Beyond efficacy and tolerability, novel agents also appear beneficial for patients with comorbid conditions like anxiety disorders or chronic pain, where these agents provide greater symptom relief compared to SSRIs and SNRIs. This superior therapeutic effect may stem from their broader and more targeted mechanisms of action, addressing overlapping neurological pathways. Moreover, the reduced drug interactions and improved side-effect profiles of novel agents make them particularly well-suited for patients with multiple chronic conditions.

Furthermore, the research offers valuable perspectives on the practical implementation of novel agents in clinical settings [19]. The potential of the novel agents as a first-line or second-line treatment for patients with major depressive disorder, especially those who have not responded well to traditional therapies, is highlighted by their ability to improve patient outcomes in terms of both depression severity and QoL, as well as their more favorable side-effect profile [20]. Clinical practice may be significantly impacted by this, providing medical professionals with a more efficient and well-tolerated therapy alternative that could eventually increase patient satisfaction and long-term adherence [21]. Better-tolerated medications may also promote longer treatment regimens, which is important in managing chronic diseases like depression, according to the novel agents’ group’s high adherence rate [22].

In addition to their clinical efficacy and tolerability, novel antidepressant agents appear to offer distinct advantages in addressing comorbidities often associated with major depressive disorder. Patients with coexisting conditions, such as anxiety disorders or chronic pain, reported greater symptom relief when treated with novel agents compared to SSRIs and SNRIs. This enhanced therapeutic effect could be attributed to the broader and more targeted mechanisms of action exhibited by these medications, which may address overlapping neurological pathways. Additionally, the improved side-effect profile and reduced drug interactions associated with novel agents make them particularly suitable for patients managing multiple chronic conditions.

Strengths, limitations, and future suggestions

The findings underscore the potential of novel agents as first-line or second-line treatments for major depressive disorder, particularly in cases resistant to conventional therapies. High adherence rates observed with novel agents highlight their capacity to promote sustained treatment engagement, which is vital for managing chronic conditions like depression. This study has several strengths, including its prospective cohort design, balanced sample sizes across treatment groups, and rigorous statistical analyses. These elements contribute to the study's internal validity and the reliability of its findings.

However, limitations must be acknowledged. The single-center design potentially restricts the generalizability of the results to broader populations. The 12-month follow-up period, while adequate for assessing short- to medium-term outcomes, may not fully capture the long-term efficacy and safety profiles of the treatments studied. The reliance on self-reported QoL measures introduces a risk of subjective bias, potentially impacting the accuracy of patient-reported outcomes. Furthermore, although the sample size was adequate for primary analyses, it may have been underpowered for detailed subgroup analyses, such as stratification by demographic or clinical characteristics.

Future studies should address these limitations by incorporating multi-center designs to enhance external validity, extending follow-up periods to better evaluate long-term treatment outcomes, and recruiting larger sample sizes to enable more detailed subgroup analyses. Additionally, the use of clinician-administered QoL assessments or objective biomarkers could help validate and complement self-reported measures. Finally, cost-effectiveness analyses in diverse real-world settings are essential for understanding the feasibility and sustainability of integrating novel agents into routine clinical practice.

## Conclusions

This study highlights the potential benefits of novel antidepressant agents compared to SSRIs and SNRIs in the treatment of major depressive disorder. Patients treated with novel agents demonstrated greater reductions in depression severity (HAM-D scores), improved QoL, and fewer adverse effects, which contributed to higher adherence rates. These findings suggest that novel agents could offer enhanced clinical outcomes, particularly for patients who are resistant to traditional therapies. However, the conclusions must be interpreted within the context of the study's limitations. The single-center design restricts the generalizability of the findings, and the 12-month follow-up period may not fully capture the long-term efficacy and safety profiles of the treatments. While robust statistical methods and adjustments were applied to minimize bias, the influence of residual or unmeasured confounders cannot be excluded.

To confirm these results and assess the broader applicability of novel agents, future studies should employ multi-center designs, longer follow-up periods, and larger, more diverse populations. Additionally, exploring confounders such as comorbidities, concurrent medications, and socioeconomic factors will provide a more nuanced understanding of treatment outcomes. Despite these limitations, the findings of this study underscore the potential of novel antidepressants to improve treatment outcomes in patients with depression, offering a foundation for further research and contributing to the evolving landscape of personalized treatment strategies in clinical practice.
